# WINTER WOES? IMPACT OF SNOW, SLEET, AND RAIN ON AGE-RELATED COGNITIVE DECLINE

**DOI:** 10.1093/geroni/igz038.1544

**Published:** 2019-11-08

**Authors:** Jessica M Finlay, Philippa Clarke, Anam Khan, Carina Gronlund, Robert Melendez, Suzanne Judd, Virginia Wadley

**Affiliations:** 1 Social Environment and Health, Institute for Social Research, University of Michigan, Ann Arbor, Michigan, United States; 2 Institute for Social Research, University of Michigan, Ann Arbor, Michigan, United States; 3 The University of Alabama at Birmingham, Birmingham, Alabama, United States

## Abstract

Snowfall, sleet and rain can adversely affect the mobility of older adults, with negative consequences for engagement in daily activities and socializing. This can lead to isolation and loneliness, which can negatively impact cognitive functioning. We tested whether long-term exposure to precipitation – particularly snow and cold rain (precipitation at ambient temperatures between 0°C and 10°C) – negatively impacts age-related cognitive function trajectories among a national sample of over 30,000 Americans (aged 45+) in the Reasons for Geographic and Racial Differences in Stroke study followed since 2003. Linear growth mixture models showed that living in an area with a 25% greater proportion of days with snow/rain in the past year was associated with a 0.6 unit decrease in cognitive function score (p<.001). Effects were stronger among those aged 75+, who experienced faster rates of cognitive decline. The findings motivate further research on the role of cold-season precipitation for cognitive decline.

